# Current Usefulness of Transesophageal Echocardiography in Patients Undergoing Transcatheter Aortic Valve Replacement

**DOI:** 10.3390/jcm12247748

**Published:** 2023-12-18

**Authors:** Jose Alberto de Agustin, Eduardo Pozo Osinalde, Carmen Olmos, Patricia Mahia Casado, Pedro Marcos-Alberca, María Luaces, Jose Juan Gomez de Diego, Luis Nombela-Franco, Pilar Jimenez-Quevedo, Gabriela Tirado-Conte, Luis Collado Yurrita, Antonio Fernandez-Ortiz, Julian Perez-Villacastin

**Affiliations:** 1 Unidad de Imagen Cardiaca, Hospital Clínico San Carlos, 28040 Madrid, Spain; eduardopozoosinalde@yahoo.es (E.P.O.); patmahia@gmail.com (P.M.C.); pedro.marcosalberca@salud.madrid.org (P.M.-A.); marialuacesm@gmail.com (M.L.); josejgd@gmail.com (J.J.G.d.D.); 2 Cardiovascular Institute, Hospital Clínico San Carlos, 28040 Madrid, Spain; luisnombela@yahoo.com (L.N.-F.); patropjq@gmail.com (P.J.-Q.); gabrielatirado@gmail.com (G.T.-C.); antonio.fernandezortiz@salud.madrid.org (A.F.-O.); julian.perez-villacastin@salud.madrid.org (J.P.-V.); 3 Department of Medicine, Universidad Complutense de Madrid, 28040 Madrid, Spain; lcollado@ucm.es

**Keywords:** aortic stenosis, transesophageal echocardiography, transcatheter aortic valve replacement

## Abstract

This review article describes in depth the current usefulness of transesophageal echocardiography in patients who undergo transcatheter aortic valve replacement. Pre-intervention, 3D-transesophageal echocardiography allows us to accurately evaluate the aortic valve morphology and to measure the valve annulus, helping us to choose the appropriate size of the prosthesis, especially useful in cases where the computed tomography is not of adequate quality. Although it is not currently used routinely during the intervention, it remains essential in those cases of greater complexity, such as for patients with greater calcification and bicuspid valve, mechanical mitral prosthesis, and “valve in valve” procedures. Three-dimensional transesophageal echocardiography is the best technique to detect and quantify paravalvular regurgitation, a fundamental aspect to decide whether immediate valve postdilation is needed. It also allows to detect early any immediate complications such as cardiac tamponade, aortic hematoma or dissection, migration of the prosthesis, malfunction of the prosthetic leaflets, or the appearance of segmental contractility disorders due to compromise of the coronary arteries ostium. Transesophageal echocardiography is also very useful in follow-up, to check the proper functioning of the prosthesis and to rule out complications such as thrombosis of the leaflets, endocarditis, or prosthetic degeneration.

## 1. Introduction

Aortic stenosis is a worldwide epidemic. New transcatheter aortic valve replacement (TAVR) techniques have shown to be a safe and effective option, compared to surgical aortic valve replacement. Candidate patients for TAVR must be assessed using different cardiovascular imaging modalities before, during, and after the procedure, among which the transesophageal echocardiogram (TEE) stands out as one of the most useful tools. An advantage of TEE over other imaging techniques, such as multidetector computed tomography, is the possibility of it being performed in real time in the hemodynamics room to guide the implantation of the prosthesis, as well as to evaluate immediate complications resulting from the procedure such as annulus rupture, pericardial effusion, or perivalvular leak. The aim of this chapter is to describe the usefulness of TEE in the different phases of the procedure.

## 2. Assessment Prior to the Intervention

**Figure 1:**
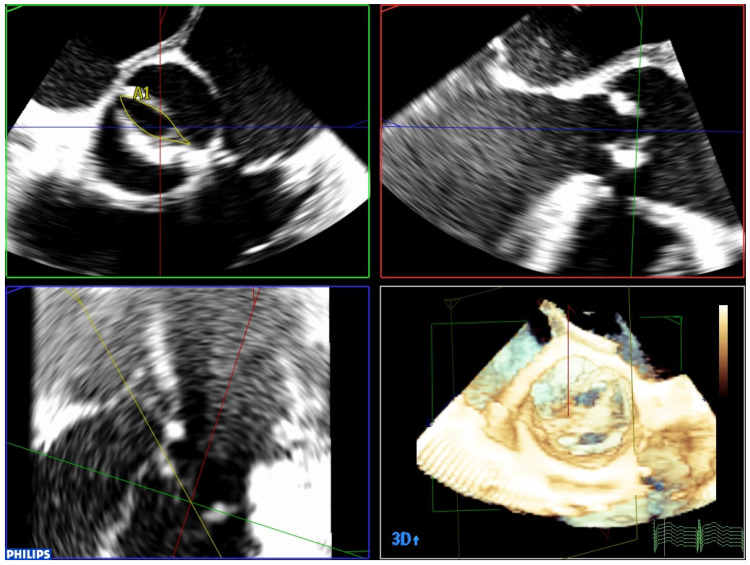
Three-dimensional TEE image using MPR mode in a patient with a bicuspid aortic valve. It makes it possible to perfectly visualize the existence of only two leaflets, as well as accurately measure the valve opening area.

TAVR includes the use of different invasive and non-invasive imaging techniques to establish the essential anatomical requirements of the valve and guide the procedure. TEE plays an important role prior to TAVR implantation. Firstly, in cases of suboptimal transthoracic window and very calcified and unstructured valves it is not easy to determine the number of leaflets. Three-dimensional TEE (3D-TEE) allows us to clarify the etiology of the disease (bicuspid or not), which may represent a relative contraindication for the procedure (Figure 1, Videos S1 and S2). TEE plays an important role in the identification of concomitant hypertrophic cardiomyopathy or conditions (such as papillary muscle anteroposition) resulting from the risk of post-procedural dynamic left ventricular obstruction.

**Figure 2:**
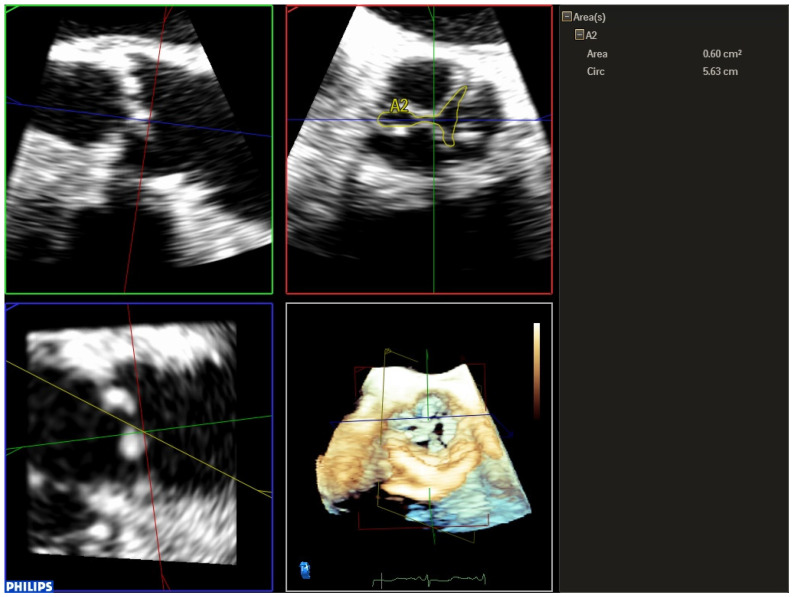
Measurement of the aortic valve area by 3D planimetry in a patient with a trileaflet aortic valve.

**Figure 3:**
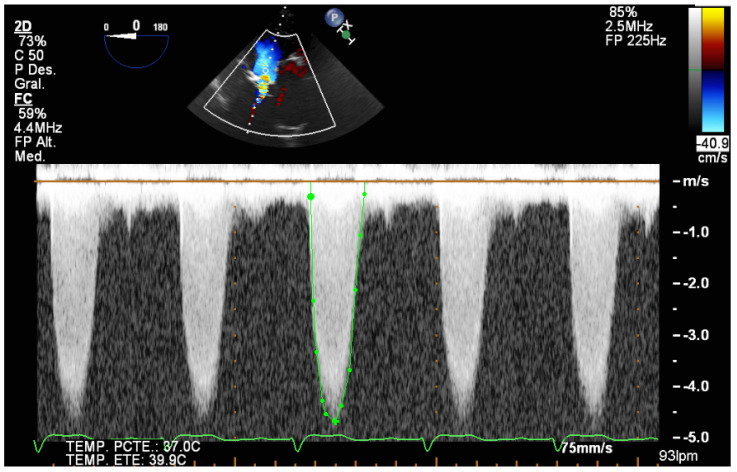
Aortic transvalvular gradients obtained from deep within the transgastric plane.

Three-dimensional TEE is also useful in cases of clinical–echocardiographic discrepancies, such as in situations of severe low-gradient aortic stenosis where there are doubts about the severity of the stenosis. The higher quality of the TEE images provides a more accurate assessment of the degree and extent of valvular calcification, the mobility of the leaflets, and the degree of valve opening than transthoracic echocardiography (Video S3). The planimetry of the orifice using TEE is more precise than if it is performed using transthoracic echo, and in this case 3D-TEE is especially useful. Three-dimensional technology allows data pyramids to be obtained in the 30° × 60° scan sectors. Post-processing allows the reconstruction of any plane and angle of the pyramidal volume previously obtained, and to obtain the valve opening area at the level of the valve cusps, whether it is a trileaflet or bicuspid valve (Figure 1 and Figure 2). TEE can also make it possible to obtain very reliable gradients in transgastric projection (Figure 3), as well as making a more precise measurement of the diameter of the left ventricular outflow tract (LVOT). It should also be explored whether there are mobile calcium elements that could become detached during the procedure, and their relationship with the coronary ostia, due to the risk of their occlusion during the implant. It must be ruled out that there are no additional processes, such as endocarditis, which we can identify by the presence of vegetations. The presence and severity of aortic valve regurgitation should also be reflected. A complete TEE should also include assessment of left ventricular function and the possible existence of intraventricular thrombi that could cause embolic complications during the procedure. Atheromatosis of the thoracic aorta (Video S4) can also increase embolic risk during the procedure, especially if it is transfemoral. Mitral valve function should also be assessed, and if mitral regurgitation exists, its severity should be established.

**Figure 4:**
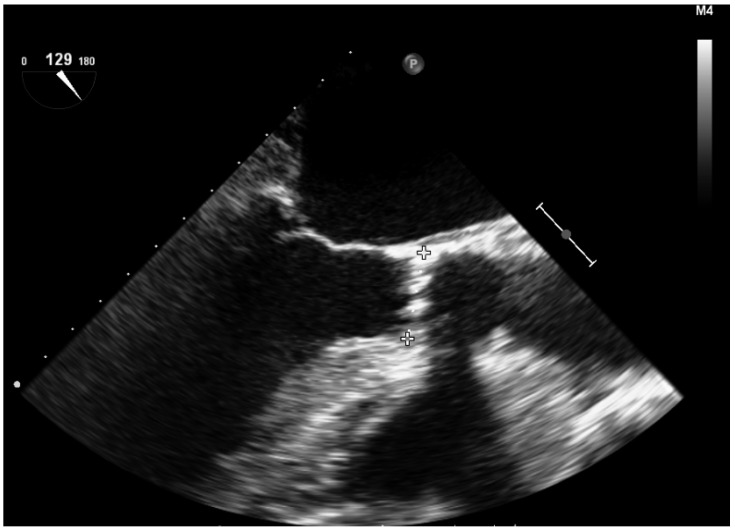
Measurement of the aortic valve annulus using 2D-TEE in the mid-esophageal plane, along the long axis of the aortic valve (120–140°).

The dimensions of the aortic annulus are a key measurement in TAVR planning, as they help select the size of valve to be used [1,2]. The measurement of the aortic annulus must be very precise, since underestimation of it implies the risk of a significant perivalvular leak or migration of the prosthesis. In case of overestimation, there is a risk of rupture of the aortic annulus, and this also increases the risk of the appearance of a conduction disorder that would require the implantation of a permanent pacemaker. Using 2D-TEE, the diameter of the aortic valve annulus is obtained in the longitudinal mid-esophageal plane at 120–140° (Figure 4). In this plane, the LVOT and its alignment with the ascending aorta are visualized. Direct comparison of transthoracic echo and TEE indicates that the measurement of the aortic annulus is approximately 1 mm larger according to the TEE than the transthoracic echo.

**Figure 5:**
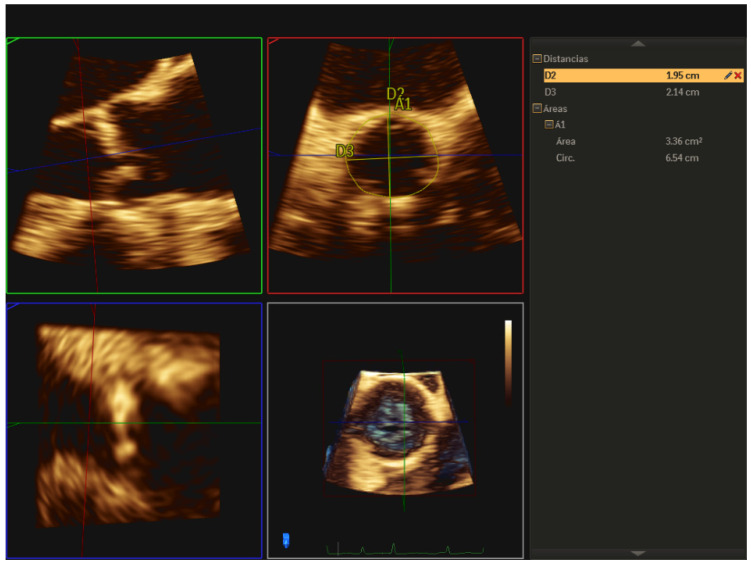
Measurement of the aortic valve annulus by 3D-TEE using the multiplanar reformatting (MPR) mode, obtaining major, minor diameters, area, and perimeter.

At present, it is recommended to measure the aortic annulus with a 3D imaging technique (multidetector computed tomography, 3D-TEE, or cardiac magnetic resonance) since the measurement obtained with a two-dimensional echocardiogram tends to systematically underestimate the size of the annulus [3]. In a series of 256 patients undergoing TAVR, annulus assessment by 3D-TEE modified the choice of prosthesis size in 23% of patients compared to if it had been based solely on 2D-TEE [4]. Similarly, measurement of the aortic annulus using 3D-TEE has been shown to reduce the incidence of perivalvular aortic regurgitation after TAVR compared to measurement with 2D-TEE [4]. For the correct measurement of the annulus using 3D-TEE, we acquire the valve in Zoom mode, and we measure the annulus in systole between the hinge points located at the junction of the leaflets with the LVOT using the multiplanar reformatting mode (MPR) (Figure 5). The 3D study of the aortic annulus makes it possible to obtain multiple measurements that include the largest and smallest diameters, the perimeter and the area, as well as the calculation of the diameters derived from the perimeter and the area. These parameters allow us to refer to the manufacturers’ tables to choose the most appropriate prosthesis size for each case. The dimensions of the annulus change throughout the cardiac cycle, so it is recommended that the acquisition of the images be carried out synchronously with the electrocardiogram and the measurements be made in systole, in the 30–40% of the RR interval, since this is the moment in the cardiac cycle during which the annulus reaches its maximum dimensions. Kasel et al. [5] proposed a novel method called the “turnaround rule” as a technique to improve annular measurements using 3D-TEE. First, the transverse and sagittal and coronal orthogonal planes are oriented along the aortic root, such that all planes intersect at the center of the opened valve, with the sagittal and coronal planes aligned parallel to the long axis of the ascending aorta. Second, the orthogonal planes are rotated to identify the most caudal attachments of the aortic valve leaflets (hinge points). The transverse plane is repositioned from the aorta toward the ventricle until it reaches the level of the hinge points. Finally, the orthogonal planes are repeatedly rotated (the turnaround rule) to ensure that the hinge points of the aortic valve leaflets are transected by the transverse plane. The annulus minimal and maximal dimeters as well as the perimeter and annular area, can then be measured on the transverse plane.

Although it is true that currently the measurement of the aortic annulus is usually performed using computed tomography, there are cases where this is not of good quality, such as in patients with rapid atrial fibrillation or in unstable patients who are unable to perform adequate apnea. There are also cases where computed tomography cannot be performed, as occurs in patients with contrast allergy or in patients where TAVR is performed urgently. In all these cases, 3D-TEE will be essential to obtain the measurement of the aortic valve annulus.

Multidetector computed tomography and 3D-TEE volumetric images have shown that the LVOT and aortic annulus have an elliptical rather than circular morphology in most patients [6]. This eccentricity of the annulus may cause the size of the selected prosthesis to be underestimated using 2D measurements, which implies a greater risk of perivalvular leak. For this reason, some studies have defined an eccentricity index as a predictor of perivalvular leak. This index is determined with the equation: 1 − (minimum diameter/maximum diameter). An eccentricity index > 0.25 has been associated with a high probability of significant perivalvular leak. This eccentricity increases in the diastolic phase [7]. Studies have also shown that the reproducibility of measuring the aortic valve annulus is higher with multidetector computed tomography and 3D-TEE than with transthoracic echo and angiography [8]. Due to the limitations of 2D-TEE, which tends to underestimate the size of the aortic annulus, in centers where the implant is guided by this technique some authors have recommended oversizing the size of the prosthesis by 1–2 mm. In the same line of thought, Mylotte et al. advise oversizing by 8–20% for balloon-expandable prostheses (Edwards SAPIEN), and 5–15% with self-expandable prostheses (CoreValve) using 2D-TEE [9].

Since TAVR placement procedures began to be performed, there has been controversy regarding which imaging technique is more accurate for measuring the aortic annulus: 3D-TEE or multidetector computed tomography. According to various studies, multidetector computed tomography is somewhat more precise and therefore the best technique for pre-procedural evaluation. This has motivated some authors, such as those of the recent Consensus Document of the American College of Cardiology on decision making in patient candidates for TAVR, not to recommend the routine use of 3D-TEE prior to the TAVR procedure [10]. 

## 3. Procedure Guide

Before the procedure, the degree of aortic and mitral regurgitation, ventricular function, the presence of pericardial effusion, and complicated atherosclerosis in the aorta must be carefully assessed, in order to compare whether complications appear during or after the procedure.

The use of TEE during the procedure (Videos S5 and S6) reduces the need for radiation exposure and the use of contrast, and allows rapid detection of complications and decision-making in complex situations that require immediate treatment. TEE is very useful, since it compensates for the lack of visualization of the tissues that fluoroscopy presents, especially when the aortic valve is poorly calcified. TEE allows us to assess, in real time, the result of the implant, the position in which the prosthesis remains and the dynamics of the leaflets (Video S7), as well as the appearance of complications. The 3D-TEE has advantages over the 2D-TEE since it allows better visualization of the guide (Video S8), the catheter, and the valve [11]. It also allows monitoring the valvuloplasty previous to the prosthesis deployment (Video S9), the correct positioning of the prosthesis (Video S10), and its deployment (Video S11).

The appropriate view for monitoring the procedure is generally a three-chamber longitudinal midesophageal plane, at 110–135°. Monitoring includes the following steps:

- Confirm the proper position of the guide and the balloon through the aortic valve (Videos S5 and S8);
- Confirm cessation of valve movement during high-frequency pacing;
- Confirm the correct position of the prosthesis prior to inflation. However, it is not always easy to identify the ends of the prosthesis when it is still retracted on the balloon. The 3D mode will help in the correct identification of its ends (Video S10). If the prosthesis is too low, it can affect the mitral valve, and if it is too high, it can migrate distally or cause coronary obstruction. It should be taken into account that during inflation the prosthesis usually moves about 3 mm in the aortic direction.

In some laboratories, the moment at which the previous valvuloplasty is performed is used to measure the maximum diameter of the balloon and corroborate the size of prosthesis that had been chosen. This measurement can be performed by both echocardiography and fluoroscopy. The combination of TEE and fluoroscopy is helpful in optimizing valve positioning and deployment. The multiplanar image allows a better observation of the transcatheter aortic valve implant in two simultaneous orthogonal planes in real time, a fact that is especially important to define the coaxiality of the prosthesis with the axis of the aorta during the implantation (Video S8). After the expansion of the prosthesis, 3D-TEE allows us to determine if the implant area is correct and the relationship between the valve support and the origin of the left coronary artery [12].

## 4. Post-Procedure Evaluation

Immediately after the procedure, the position of the prosthesis must be verified (normally implanted, low implant in the LVOT or high implant in the aorta), along with the degree of intravalvular or perivalvular leak, the degree of mitral regurgitation, ventricular function, and the presence and degree of pericardial effusion. If the prosthesis is positioned too low, it may affect the mitral valve apparatus, or it may be difficult to stabilize in patients with severe hypertrophy of the basal interventricular septum. On the contrary, if the prosthesis is implanted too high, it can migrate towards the aorta, obstruct the ostium of the coronary arteries, or cause significant perivalvular regurgitation.

What are the criteria for adequate implementation?

In the evaluation in the short axis at the level of the great vessels, the prosthesis must have a circular and not ovoid shape, and in the long parasternal axis, the proximal edge must remain a few millimeters inside the LVOT, without having excessive protrusion. It should have a peak velocity ≤ 2 m/s, and no significant perivalvular (Video S12) or transvalvular leak should be observed. If one or more of these data are not met, repositioning the prosthesis could be considered, especially for self-expanding ones, or postdilatation of the prosthesis with a balloon (Video S13). Previous studies have shown that predilation decreases the need for postdilation immediately after valve deployment [13]. However, predilatation increases the risk of strokes, displacement of the prosthesis after deployment, conduction disturbances requiring the implantation of a permanent pacemaker, and even annular rupture [14]. In addition, postdilation is also a known predictor of acute cerebrovascular events and is associated with increased 30-day mortality or major stroke. Therefore, nowadays, it remains controversial whether it is better to perform predilation. This is usually performed in self-expanding prostheses, which have more difficulty deploying, while, in balloon-expandable prostheses, it is not usually necessary. It should also be noted that the latest generation prostheses present better results, with less perivalvular insufficiency and a reduced need for postdilation [15].

As a last option, one can consider implanting another transcatheter valve inside the previous one, using the procedure called “valve-in-valve” (Videos S14–S16).

In several series, up to 7–30% of perivalvular leaks were observed after TAVR. The factors that are related to the appearance of aortic regurgitation are low implantation of the device, a small prosthesis, and the presence of calcium in the aortic annulus. If a significant leak that is difficult to evaluate is detected immediately after implantation, any catheters that may still be passing through the valve should be removed to rule out an intraprosthetic leak caused by the catheter or guide. According to the VARC II (Valve Academic Research Consortium), the semiquantitative parameters for the evaluation of perivalvular leaks are: reverse diastolic flow in the descending aorta (absent = mild; intermediate = moderate; prominent or holodiastolic = severe). The most widespread way to quantify perivalvular regurgitation is by its circumferential extension in the aortic annulus (<10% = mild; 10–20% = moderate; ≥30% = severe). The established quantitative parameters are [16]: regurgitant volume (<30 mL = mild; 30–59 mL = moderate; ≥60 mL = severe), regurgitant fraction (<30% = mild; 30–49% = moderate; ≥50% = severe), and effective regurgitant orifice area (ORE) (<0.10 cm^2^ = mild; 0.10–0.29 cm^2^ = moderate; ≥0.30 cm^2^ = severe) [17].

**Figure 6:**
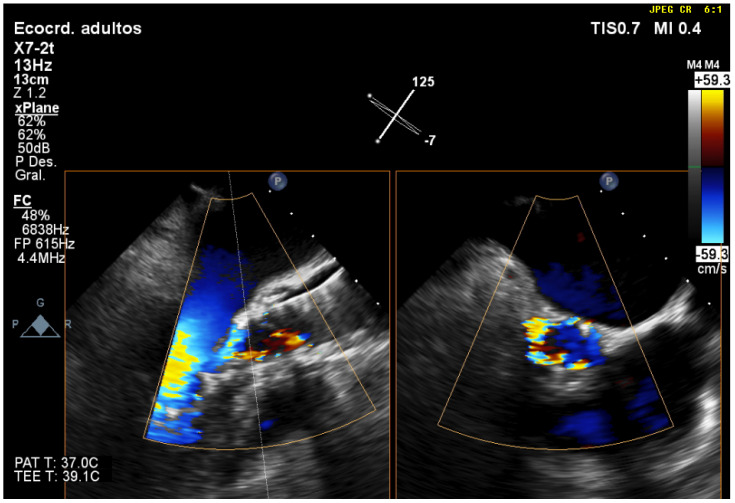
Evaluation of post-TAVR perivalvular aortic regurgitation using 3D-TEE in biplanar mode (X-plane). In the short axis, it is observed that regurgitation covers more than 30% of the circumference, which corresponds to severe regurgitation.

Three-dimensional TEE makes it possible to quantify aortic regurgitation more precisely. Visualization of two orthogonal planes with the biplanar color method localizes the jet and evaluates the mechanism and severity of aortic regurgitation more accurately than 2D-TEE (Figure 6 and Video S17). Planimetry of the vena contracta area by 3D-TEE facilitates a better classification of moderate regurgitation [18]. Another possible complication of the procedure is embolization of the prosthesis, which is a rare and serious complication that must be diagnosed quickly (Video S18). Sometimes there is a displacement of the prosthesis towards the LVOT, which can cause mitral regurgitation due to restriction of the anterior mitral leaflet. Displacement towards the aorta can cause occlusion of the coronary ostia and, as a consequence, ventricular dysfunction. The presence of QRS widening or hemodynamic deterioration with abnormalities in segmental contractility after expansion of the prosthesis should alert physicians to possible compromise due to coronary artery obstruction. In one study, 70% of patients with coronary obstruction had a left main coronary artery height < 12 mm and a maximum aortic root diameter < 30 mm. It is also important to evaluate the aortic root, since it could have increased in thickness due to an intramural aortic hematoma (Video S19) or an aortic dissection. The sudden appearance of pericardial effusion (Video S20) should alert physicians to possible ventricular perforation or rupture of the aortic annulus.

Dynamic LVOT obstruction may be a dreadful event following TAVR. Patients with severe aortic stenosis sometimes develop asymmetric septal hypertrophy leading to LVOT obstruction. Predictors of left ventricular obstruction include small left ventricular end diastolic diameter, high ejection fractions, high intraventricular septum to posterior wall ratios, and high valve gradients. Before the procedure, evidence of LVOT obstruction via both symptoms and echocardiographic findings may be minimized due to extremely high afterload on the left ventricle. With the abrupt decrease in afterload after placement of the new valve, a LVOT obstruction may develop. Diagnosing a LVOT obstruction as the cause of hemodynamic instability during TAVR, in the absence of abnormal findings on echocardiogram preoperatively, requires a high index of clinical suspicion, and TEE is essential to detecting it early. The management of acute onset of a LVOT obstruction intraoprocedurally consists primarily of medical therapy, including rate control, adequate volume resuscitation, and avoidance of inotropes. With persistently elevated gradients, procedural interventions should be considered based on individual patients’ surgical risk, and myomectomy or urgent septal alcohol ablation may be considered.

For all the reasons stated above, the usefulness of 3D-TEE is beyond doubt in the evaluation and selection of patients who are candidates for TAVR, as well as in adequately determining the size of the device, correctly guiding implantation of the prosthesis, evaluating the result, and detecting immediate complications stemming from the procedure. However, despite all these advantages, its use requires complete anesthesia and orotracheal intubation of the patient. 

Although TEE is a noninvasive diagnostic technique that is considered relatively safe across a broad spectrum of patients, severe life-threatening complications have been reported [19]. The insertion and manipulation of the ultrasound probe can cause oropharyngeal, esophageal, or gastric trauma. Mechanical injury to the esophageal and gastric body can lead to life-threatening bleeding. The estimated risk of significant gastrointestinal bleeding is 0.02% and 1.0%. Patients with friable mucosal tissue from underlying disease are at higher risk. Anesthetic strategies vary in different centers. Local anesthesia or general anesthesia are both valid alternatives and can be applied according to the patient’s characteristics and procedural instances. General anesthesia offers many advantages, mainly regarding the possibility of an early diagnosis and treatment of possible complications through the use of transesophageal echocardiography. 

The evolution of the technique, which is increasingly performed more safely and with fewer complications, has called into question the need to systematically perform control with TEE and general anesthesia in all patients and has led to the proposal of a “minimalist” protocol, with transfemoral access, conscious sedation, and subsequent control with transthoracic echo. There are already some publications that have shown that this protocol, carried out in experienced and high-volume centers, is safe and is associated with simpler subsequent patient management, with earlier mobilization, shorter hospital stays, and lower costs. The subsequent and current trend is towards moderate sedation, without endotracheal intubation and guided by transthoracic echocardiogram [20]. In some situations, such as for patients with mechanical mitral prosthesis, where TAVR may compromise the movement of some of the discs, or also in “valve-in-valve” procedures, it is still essential to perform TEE during the procedure. Also, in cases where the multidetector computed tomography images are not of optimal quality and there are doubts about the size of the prosthesis to be implanted, it is recommended to measure the annulus immediately before the procedure using 3D-TEE.

## 5. Follow-Up

TEE is also a very useful test in follow-up, to check the proper functioning of the prosthesis and rule out complications such as thrombosis of the leaflets, endocarditis, or prosthetic degeneration. Although bioprosthetic valves are less thrombogenic than their mechanical counterparts, valve thrombosis is an important issue [21]. Clinical valve thrombosis is defined as prosthetic valve dysfunction with the typical finding of a thrombus on the valve accompanied with an elevated transvalvular pressure gradient and often symptoms of heart failure or left-sided thromboembolic events. Clinical valve thrombosis has been reported to be relatively rare after TAVR, with an incidence of 0.6–2.8%, and OAC may resolve the thrombosis and elevated gradient as well as clinical symptoms. A more common finding in both transcatheter and surgical bioprosthetic aortic valves is subclinical leaflet thrombosis, with a thin layer of thrombosis on the aortic side of one or more of the bioprosthetic leaflets. This phenomenon is best visualized on four-dimensional volume-rendered computed tomography. The clinical relevance of subclinical leaflet thrombosis is also still unclear in terms of association with thrombo-embolic events.

TEE is the first-choice imaging technique in the diagnostic workup of suspected infective endocarditis, making it possible to detect the presence of vegetations, abscesses, and pseudoaneurysms. Importantly, infective endocarditis should always be suspected in patients with new periprosthetic regurgitation until proven otherwise. Three-dimensional TEE is of incremental value for the analysis of vegetation morphology and size.

TEE is the best imaging technique to detect prosthetic degeneration. According to the VARC-3 definitions, transcatheter valve degeneration is defined as intrinsic permanent changes to the prosthetic valve (which may include wear and tear, leaflet disruption, flail leaflet, fibrotic leaflet/calcification, or strut fracture) with an increase in transvalvular gradient ≥ 20 mmHg, resulting in a mean gradient ≥ 30 mmHg with a concomitant decrease in effective orifice area ≥ 0.6 cm^2^ (or ≥50% of prior EOA), compared with echocardiographic assessment performed 1 to 3 months postprocedure, or with a new occurrence or increase by ≥2 grades of intraprosthetic aortic regurgitation resulting in severe aortic regurgitation.

## 6. Future Directions

The future of TEE in TAVR involves the introduction of new microprobes with all the capabilities of those we normally use (biplanar imaging, 3D imaging, etc.). Until now, existing microprobes had very limited functions (only 2D imaging and low-quality color). Interventional procedures are evolving towards the lowest levels of invasiveness possible, and for this purpose it is essential to avoid deep sedation and orotracheal intubation. The new microprobes that are already available make it possible for the procedure to be perfectly monitored without the need of orotracheal intubation and with minimal discomfort for the patient. Additionally, these new probes come equipped with all the necessary capabilities that the microprobes we had in the past lacked. 

Another possible way to perform pre-procedures in a non-invasive way is through non-invasive ventilation using the Janus mask [22]. The Janus mask has a hole that allows TEE examination during non-invasive ventilation, and it can be opened (and closed) around the endoscopic probe, facilitating insertion of the TEE probe into the patient’s mouth.

**Figure 7:**
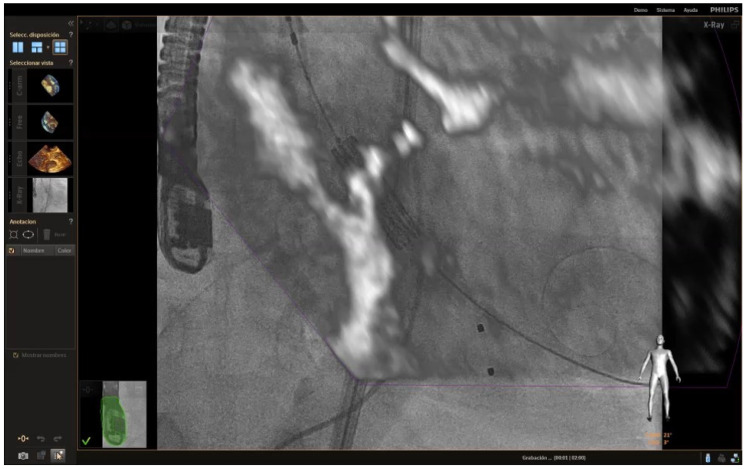
Fusion imaging showing the TAVR just before its deployment at the level of the aortic annulus.

Another great advance that is coming is new fusion techniques, which make it possible the fluoroscopy and the echocardiogram image to be seen simultaneously superimposed on the screen (Figure 7). This tool allows procedures to be monitored with greater security and makes it possible to reduce the amount of contrast used. The combination of these two great advances will provide the opportunity to do the procedures much faster and more safely for the patient.

## 7. Conclusions

TEE is the ideal imaging technique for the assessment of multiple aspects of a patient who is going to undergo TAVR. Although other techniques, such as magnetic resonance imaging or multidetector computed tomography, can provide relevant information, echocardiography plays an important role in patient selection, in monitoring the procedure, and in the immediate detection of complications. Take-home messages are described in Table 1.

## Figures and Tables

**Table 1:** Take-home messages.

1	Three-dimensional TEE allows us to accurately evaluate the aortic valve morphology and measure the valve annulus prior to TAVR implantation, helping us to choose the appropriate size of the prosthesis, especially useful in cases where the cardio-CT is not of adequate quality.
2	TEE monitoring during TAVR implantation allows us to properly position the valve in the aortic annulus, avoiding positioning errors and possible migrations of the prosthesis.
3	Three-dimensional TEE is the best technique to detect and quantify perivalvular regurgitation after TAVR implantation, a fundamental aspect in deciding whether immediate valve postdilation is needed or not.
4	TEE allows us to early detect complications during the procedure such as cardiac tamponade, hematoma or aortic dissection, migration of the prosthesis, or malfunction of any of the prosthetic leaflets.
5	Three-dimensional TEE allows us to accurately evaluate cardiac function after TAVR, with important aspects such as systolic function or mitral valve function, which can be affected by TAVR when the implant is low, or due to damage caused to the subvalvular apparatus by the catheters and guides.
6	The combination of the new microprobes and the new fusion techniques, which allow the fluoroscopy and the echocardiogram image to be seen simultaneously, will provide the opportunity to do the procedures much faster and more safely for the patient.

## Data Availability

Not applicable.

## Supplementary Materials

The following supporting information can be downloaded at: https://www.mdpi.com/article/10.3390/jcm12247748/s1.
